# 布鲁顿酪氨酸激酶抑制剂单药治疗初诊华氏巨球蛋白血症患者的疗效和安全性

**DOI:** 10.3760/cma.j.issn.0253-2727.2023.06.008

**Published:** 2023-06

**Authors:** 怡 陶, 芸璐 徐, 硕 王, 黎 王, 维莅 赵

**Affiliations:** 上海血液学研究所，医学基因组学国家重点实验室，国家转化医学中心（上海），上海交通大学医学院附属瑞金医院，上海 200025 Shanghai Institute of Hematology, State Key Laboratory of Medical Genomics, National Research Center for Translational Medicine at Shanghai, Ruijin Hospital Affiliated to Shanghai Jiao Tong University School of Medicine, Shanghai 200025, China

**Keywords:** Waldenstrom巨球蛋白血症, 布鲁顿酪氨酸激酶抑制剂, 治疗结果, 不良反应, Waldenstrom Macroglobulinemia, Bruton's tyrosine kinase inhibitor, Treatment outcome, Adverse reaction

## Abstract

**目的:**

探索布鲁顿酪氨酸激酶抑制剂（BTKi）伊布替尼或泽布替尼单药治疗初诊华氏巨球蛋白血症（WM）患者的疗效和安全性。

**方法:**

回顾性分析2018年1月至2022年8月于上海交通大学医学院附属瑞金医院接受BTKi单药治疗的58例初诊WM患者的疗效和安全性。

**结果:**

55例患者可评估疗效，40例患者接受伊布替尼单药治疗，中位治疗时间15个月，总缓解率（ORR）为85％，主要缓解率（MRR）为70％，非常好的部分缓解（VGPR）率为10％。15例患者接受泽布替尼单药治疗，中位治疗时间13个月，ORR为93％，MRR为73％，VGPR率为0％。10例患者由于各种原因由伊布替尼转换为泽布替尼治疗，转换前伊布替尼中位用药时间7.5个月，转换后泽布替尼中位用药时间3.5个月，转换前、后的ORR分别为90％和100％，MRR分别为80％和80％，VGPR率分别为10％和50％。中位随访时间为16个月，接受两种BTKi治疗患者预期24个月PFS率均为86％，ISS评分低、中、高危患者PFS的差异无统计学意义（*P*＝0.998），所有患者均存活。BTKi不良反应主要表现为中性粒细胞减少和血小板减少，发生率分别为12％和10％。伊布替尼组心房颤动的发生率为5％，泽布替尼组出血的发生率为7％。

**结论:**

伊布替尼或泽布替尼单药治疗WM均有良好的疗效和可耐受的不良反应。

## Abstract (en)

**Objective:**

To investigate the efficacy and safety of Bruton tyrosine kinase inhibitors (BTKi) ibrutinib or zanubrutinib monotherapy in newly diagnosed patients with Waldenström macroglobulinemia (WM).

**Methods:**

The efficacy and adverse effects of 58 patients with newly diagnosed WM receiving BTKi monotherapy in Ruijin Hospital Affiliated to Shanghai Jiao Tong University School of Medicine were analyzed retrospectively from January 2018 to August 2022.

**Results:**

The response of 55 patients may be examined. Forty patients received ibrutinib monotherapy for a median of 15 months, with an overall response rate (ORR) of 85％, a main remission rate (MRR) of 70％, and a very good partial remission (VGPR) rate of 10％. Fifteen patients received zanubrutinib monotherapy for a median of 13 months, with an ORR of 93％, an MRR of 73％, and a VGPR rate of 0％. For various reasons, 10 patients were converted from ibrutinib to zanubrutinib. Ibrutinib treatment lasted an average of 7.5 months before conversion. The median duration of zanubrutinib therapy after conversion was 3.5 months. The ORRs before and after conversion were 90％ and 100％, MRRs were 80％ and 80％, and VGPR rates were 10％ and 50％, respectively. After a median of 16 months, the 24-month progression-free survival (PFS) rate of patients who received both BTKi was 86％. PFS did not differ statistically across individuals with low, medium, and high-risk ISS scores (*P*＝0.998). All of the patients survived. The most common side effects of BTKi were neutropenia and thrombocytopenia, which occurred in 12％ and 10％ of all patients, respectively. Ibrutinib accounts for 5％ of atrial fibrillation, and zanubrutinib has a 7％ risk of bleeding.

**Conclusion:**

In treating WM, ibrutinib or zanubrutinib provides good efficacy and tolerable adverse effects.

华氏巨球蛋白血症（WM）是一种罕见的分泌IgM的惰性淋巴浆细胞淋巴瘤，在非霍奇金淋巴瘤中占2％[1]。>90％和30％～35％的WM患者分别发现髓样分化因子88（MYD88）和趋化因子受体4（CXCR4）的体细胞突变[2]。MYD88是白细胞介素-1和Toll样受体信号传导复合物的组成成分，MYD88^L265P^突变激活造血细胞激酶（HCK），HCK进一步激活布鲁顿酪氨酸激酶（BTK），最终活化核转录因子NF-κB促进淋巴浆细胞的恶性增殖[3]。Treon等[4]和Trotman等[5]分别报道了伊布替尼单药和泽布替尼单药在WM患者中的长期随访结果。中国初诊WM患者应用BTK抑制剂（BTKi）单药伊布替尼或泽布替尼治疗的研究尚无报道。本研究回顾性分析58例接受伊布替尼或泽布替尼单药治疗的初诊WM患者，初步探索这两种BTKi的疗效及不良反应。

## 病例与方法

1. 病例：回顾性分析2018年1月至2022年8月上海交通大学医学院附属瑞金医院符合治疗指征并接受BTKi单药治疗的58例初诊WM患者。所有患者符合以下WM诊断标准[6]–[7]：①血清中存在单克隆IgM（不论数量）；②病理检查证实骨髓中存在淋巴浆细胞浸润；③除外其他已知类型的淋巴瘤。

2. 危险分层：采用WM国际预后评分系统（ISSWM）进行危险分层[8]。该预后系统纳入5个危险因素，即年龄>65岁，HGB≤115 g/L，PLT≤100×10^9^/L，β_2_-微球蛋白（β_2_-MG）>3 mg/L，IgM>70 g/L。低危组：0或1个危险因素且年龄≤65岁；中危组：2个危险因素或年龄>65岁；高危组：2个以上危险因素。

3. MYD88^L265P^和CXCR4突变检测：方法包括一代测序（Sanger）、等位基因特异性聚合酶链反应（AS-PCR）和二代测序（NGS），标本来源包括淋巴结、骨髓和外周血。进行MYD88^L265P^突变检测的52例患者中，51例（98％）患者采用NGS和（或）AS-PCR方法，47例（90％）患者采用骨髓和（或）淋巴结。骨髓和淋巴结肿瘤组织中基因组DNA通过DNA纯化试剂盒（美国QIAGEN公司产品）提取并定量。外周血和骨髓未经CD19分选。标本留取获得患者知情同意。

4. 疗效和疾病进展的定义：58例初诊患者均接受BTKi单药治疗，其中有43例接受伊布替尼治疗口服420 mg，每日1次；15例接受泽布替尼治疗口服160 mg，每日2次。采用第7届国际WM工作组（IWWM）制定的标准定义疗效和疾病进展[9]。总缓解率（ORR）定义为至少获得微小缓解（MR）的患者比例，主要缓解率（MRR）定义为至少获得部分缓解（PR）的患者比例。无进展生存（PFS）时间定义为自患者开始治疗至疾病进展或死亡的时间，总生存（OS）时间定义为自患者开始治疗至死亡或末次随访的时间。不良反应的评价标准参考CTCAE 5.0。

5. 伊布替尼转换为泽布替尼患者：10例初诊患者接受伊布替尼治疗后转换为泽布替尼治疗，2例患者不耐受，其中1例表现为出血，1例表现为乏力伴皮疹；2例是MYD88野生型；2例是MYD88和CXCR4双突变；2例疗效未达PR；2例MYD88突变CXCR4野生型患者获得PR后希望追求更深程度的缓解。10例转换治疗患者转换治疗前的最佳疗效和预后情况纳入伊布替尼组，由于此10例患者非初诊接受泽布替尼治疗，因此未纳入泽布替尼组。

6. 随访：通过查看门诊记录、住院病历及电话咨询的方式随访，末次随访日期为2022年10月23日。中位随访时间16（1～55）个月。

7. 统计学处理：采用SPSS 23.0软件进行统计学分析。计数资料用例数（百分比）表示，计量资料用中位数（范围）表示。组间率的比较采用似然比*χ*^2^检验，采用Kaplan-Meier法分析患者的生存情况，*P*<0.05为差异有统计学意义。

## 结果

1. 临床特征：58例初诊WM患者的基线临床特征见表1。

**表1:** 接受伊布替尼或泽布替尼单药治疗的初诊华氏巨球蛋白血症患者的临床特征

临床特征	总体（58例）	伊布替尼组（43例）	泽布替尼组（15例）
年龄[岁，*M*（范围）]	68（42～88）	68（47～88）	68（42～86）
年龄>65岁[例（%）]	35（60）	25（58）	10（67）
性别[例（%）]			
男	49（84）	35（81）	14（93）
女	9（16）	8（19）	1（7）
ISS评分[例（%）]			
低危	12（21）	10（23）	2（13）
中危	22（38）	18（42）	4（27）
高危	24（41）	15（35）	9（60）
IgM[g/L，*M*（范围）]	37.30（4.55～117.74）	33.60（4.55～117.74）	47.64（4.70～113.26）
IgM>30 g/L[例（%）]	35（60）	23（53）	12（80）
HGB[g/L，*M*（范围）]	94（53～163）	94（53～163）	93（63～142）
HGB<110 g/L[例（%）]	39（67）	29（67）	10（67）
PLT[×10^9^/L，*M*（范围）]	174（26～589）	175（26～589）	172（34～379）
PLT<100×10^9^/L[例（%）]	12（21）	9（21）	3（20）
血清β_2_-MG[mg/L，*M*（范围）]	2.6（0.1～18.1）	2.5（0.1～18.1）	3.1（1.5～7.9）
血清β_2_-MG>3 mg/L[例（%）]	24（41）	15（35）	9（60）
骨髓受累[%，*M*（范围）]	9（1～73）	7（1～73）	13（2～67）
髓外受累[例（%）]			
淋巴结最大直径>1.5 cm^a^	21（60）	14（54）	7（78）
脾脏长径>15 cm^b^	5（15）	4（17）	1（11）
MYD88突变^c^[例（%）]	46（88）	35（85）	11（100）
CXCR4突变^d^[例（%）]	10（24）	9（26）	1（14）

注 β_2_-MG：β_2_-微球蛋白；^a^共35例患者有此项数据；^b^共33例患者有此项数据；^c^共52例患者进行了MYD88突变检测；^d^共42例患者进行了CXCR4突变检测

2. 疗效：排除3例患者基线IgM<5 g/L，有55例患者可评估疗效，其中伊布替尼组40例，泽布替尼组15例。所有患者中位接受BTKi治疗15（1～55）个月，ORR为87％，MRR为71％，非常好的部分缓解（VGPR）率为7％。其中伊布替尼组中位治疗时间15（1～55）个月，ORR为85％，MRR为70％，VGPR率为10％。泽布替尼组中位治疗时间13（1～30）个月，ORR为93％，MRR为73％，VGPR率为0％。根据患者MYD88和CXCR4基因突变情况（41例）进行分组，MYD88突变CXCR4野生型（MYD88^MUT^CXCR4^WT^）组（26例）MRR为73％，其中伊布替尼组（20例）MRR为70％，泽布替尼组（6例）MRR为83％。MYD88突变CXCR4突变型（MYD88^MUT^CXCR4^MUT^）组（9例）MRR为67％，其中伊布替尼组（8例）MRR为63％，泽布替尼组（1例）MRR为100％。MYD88野生型（MYD88^WT^）组（6例）MRR为67％，均为伊布替尼组，泽布替尼组患者未检测到MYD88^WT^。

3. 由伊布替尼转换为泽布替尼治疗患者的疗效：10例患者由于各种原因由伊布替尼单药治疗转换为泽布替尼单药治疗，接受伊布替尼单药中位治疗时间7.5个月，转换后泽布替尼中位治疗时间3.5个月，转换前、后的ORR分别为90％和100％，MRR分别为80％和80％，VGPR率分别为10％和50％。

4. 预后：所有使用BTKi单药治疗患者中位随访16个月，24个月PFS率为86％。其中伊布替尼组和泽布替尼组中位随访时间均为16个月，24个月PFS率均为86％，所有患者均存活。ISS评分低、中、高危患者PFS的差异无统计学意义（*P*＝0.998）（图1）。在ISS高危患者中比较接受两种BTKi治疗患者的PFS，差异无统计学意义（*P*＝0.371）。

**图1:**
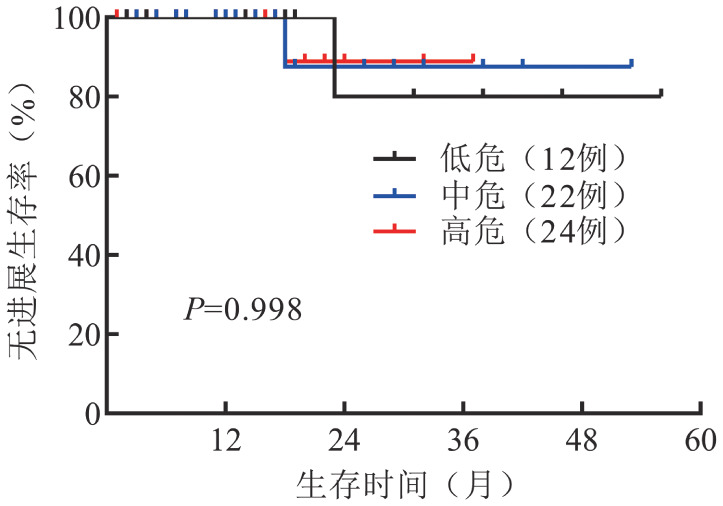
ISS评分低、中、高危华氏巨球蛋白血症患者的无进展生存曲线

5. 不良反应：BTKi单药引起中性粒细胞减少的总发生率为12％（7/58），其中伊布替尼组为12％（5/43），泽布替尼组为13％（2/15），均未见3级及以上中性粒细胞减少。BTKi单药引起血小板减少的总发生率为10％（6/58），其中伊布替尼组为12％（5/43），3级及以上占5％（2/43）；泽布替尼组为7％（1/15），未见3级及以上血小板减少。其他不良反应均小于3级，伊布替尼组心房颤动发生率为5％（2/43）、心动过速发生率为5％（2/43）、出血发生率为5％（2/43）、皮疹发生率为7％（3/43）；泽布替尼组心动过速发生率为7％（1/15）、出血发生率为7％（1/15）。

## 讨论

本研究回顾性分析本中心58例应用BTKi单药的初诊WM患者，根据患者的临床特征，泽布替尼组ISS高危患者比例较高，达60％，而我们的预后分析结果与Treon等[4]的报道一致，BTKi能克服高危WM患者的不良预后。因此，烷化剂或嘌呤类似物时代适用的ISS危险度分层可能不再适用于BTKi靶向药物治疗时代，需要探索新的预后分层因素。WM患者的治疗经历了以烷化剂为代表的传统药物时代、以CD20单抗或蛋白酶体抑制剂为基础的新药时代和以BTKi为主的靶向药物时代，患者的PFS和OS明显改善。在BTKi单药长期随访的临床研究中，伊布替尼单药治疗63例经治WM患者的5年PFS率和OS率分别为54％和87％[4]，泽布替尼治疗77例WM患者（24例初治，53例经治）的3年PFS率和OS率分别为80.5％和84.8％[5]。本中心既往研究报道，108例WM患者5年预估PFS率和OS率分别为28％和67％[10]，提示WM患者应用BTKi单药治疗有较好的长期预后。本研究发现，接受伊布替尼或泽布替尼单药的初诊WM患者24个月PFS率均为86％，也符合ASPEN研究中两药PFS无显著差异的结果[11]。

研究显示，伊布替尼单药治疗随访5年的MRR为78％，VGPR率为27％[4]，泽布替尼单药治疗随访3年的MRR为82％，VGPR率为45％[5]。本研究中初诊患者无论接受伊布替尼（中位用药15个月，MRR为70％）还是泽布替尼（中位用药13个月，MRR为73％），均取得良好的疗效。本研究的结果与中位随访19.4个月的ASPEN研究相似，无论是伊布替尼还是泽布替尼，均无患者达到完全缓解（CR），提示BTKi长期治疗的必要性。APSPEN研究提示，在MYD88阳性患者中，泽布替尼的VGPR率优于伊布替尼（28％对19％）[11]。APSPEN的单臂研究提示，MYD88阴性患者泽布替尼单药治疗的VGPR率为27％（中位随访17.9个月），既往在MYD88阴性患者中未报道伊布替尼的VGPR率，iNNOVATE研究联合应用伊布替尼和CD20单抗，VGPR率为27％[12]。本研究为回顾性研究，未能凸显泽布替尼在初诊患者中VGPR率的优势，原因可能与泽布替尼用药时间较短和患者例数较少有关。报道显示，泽布替尼应用24个月达到最佳平台期，提示我们在评估BTKi疗效时要关注用药时间[5]。本研究发现，10例初诊接受伊布替尼的患者在转换为泽布替尼治疗后（中位用药时间3.5个月），VGPR率由转换前的10％提高到50％。在以CD20单抗为基础的治疗时代，WM患者获得VGPR可达到与获得CR同样的PFS[13]。有报道称泽布替尼对BTK的选择性更强，脱靶效应更低[11]。也可能是泽布替尼每日2次的给药方式使血药浓度和BTK占有率更稳定，因为在淋巴结活检中观察到泽布替尼对BTK 100％的持续占有时间超过24 h[12]。值得注意的是，无论是伊布替尼还是泽布替尼，延长用药时间都能加深治疗深度，因此上述10例转换患者如果继续延长接受伊布替尼治疗的时间，其缓解程度有可能进一步改善。

本研究报道初诊WM患者MYD88^L265P^突变阳性率88％，与国际临床研究报道的比例相似[2],[14]，较既往本中心报道的突变率77％偏高[10]。本中心曾总结，在MYD88突变检测方面，骨髓液和淋巴结标本优于外周血，NGS和AS-PCR法优于Sanger法[15]。因此本研究MYD88^L265P^突变检出率增高可能由于90％初诊患者采用了骨髓液和（或）淋巴结作标本，98％患者通过NGS和（或）AS-PCR方法检测。MYD88和CXCR4的基因分型是BTKi疗效和预后的重要参考，无论是接受伊布替尼还是泽布替尼单药治疗，MYD88^MUT^CXCR4^WT^患者疗效较好，MYD88^MUT^CXCR4^MUT^其次，MYD88^WT^较差[2],[4],[14]。本中心研究显示，接受BTKi治疗患者中MYD88^MUT^CXCR4^WT^亚型MRR最高，为73％，而MYD88^MUT^CXCR4^MUT^和MYD88^WT^的MRR均为67％。Pivotal研究中经治MYD88^WT^患者接受伊布替尼治疗的MRR为28.6％[2]，ASPEN子研究中初诊和经治MYD88^WT^患者接受泽布替尼治疗的MRR为50％[12]。本研究中6例MYD88^WT^患者均接受伊布替尼治疗，MRR为67％，一方面可能由于患者为初诊且例数较少，另一方面与MYD88检测方法有关。6例MYD88^WT^中4例疗效≥PR，其中1例通过Sanger法，另1例通过外周血检测MYD88突变，通过Sanger法和外周血检测可能增加MYD88假阴性率[15]。

在不良反应方面，ASPEN研究表明，伊布替尼导致心房颤动和高血压的发生率略高，而泽布替尼导致的粒细胞减少较常见[11]。Treon等[4]跟踪接受伊布替尼单药治疗5年的患者，发现心房颤动发生率不足10％，其中3级以上占1.6％。本中心在随访中发现伊布替尼引起心房颤动的发生率为5％，泽布替尼引起出血的发生率为7％，均不足10％且无3级及以上严重不良反应，因此BTKi单药安全性良好。

WM患者的治疗进入了靶向药物时代，我们的研究发现，伊布替尼和泽布替尼治疗初诊WM患者均体现出良好的疗效和可耐受的不良反应。新一代BTKi泽布替尼在治疗深度中的优势有待进一步通过延长用药时间和积累更多患者得到证实，这种优势能否最终转换为PFS的优势也需延长随访时间。

## References

[1] Morton LM, Turner JJ, Cerhan JR (2007). Proposed classification of lymphoid neoplasms for epidemiologic research from the Pathology Working Group of the International Lymphoma Epidemiology Consortium (InterLymph)[J]. Blood.

[2] Treon SP, Tripsas CK, Meid K (2015). Ibrutinib in previously treated Waldenstrom's macroglobulinemia[J]. N Engl J Med.

[3] Yang G, Buhrlage SJ, Tan L (2016). HCK is a survival determinant transactivated by mutated MYD88, and a direct target of ibrutinib[J]. Blood.

[4] Treon SP, Meid K, Gustine J (2021). Long-Term Follow-Up of Ibrutinib Monotherapy in Symptomatic, Previously Treated Patients With Waldenström Macroglobulinemia[J]. J Clin Oncol.

[5] Trotman J, Opat S, Gottlieb D (2021). Zanubrutinib for the treatment of patients with Waldenström macroglobulinemia: 3 years of follow-up. Blood, 2020, 136(18): 2027-2037[J]. Blood.

[6] Owen RG, Treon SP, Al-Katib A (2003). Clinicopathological definition of Waldenstrom's macroglobulinemia: consensus panel recommendations from the Second International Workshop on Waldenstrom's Macroglobulinemia[J]. Semin Oncol.

[7] 中国抗癌协会血液肿瘤专业委员会, 中华医学会血液学分会, 中国华氏巨球蛋白血症工作组 (2022). 淋巴浆细胞淋巴瘤/华氏巨球蛋白血症诊断与治疗中国指南(2022年版)[J]. 中华血液学杂志.

[8] Morel P, Duhamel A, Gobbi P (2009). International prognostic scoring system for Waldenstrom macroglobulinemia[J]. Blood.

[9] Dimopoulos MA, Kastritis E, Owen RG (2014). Treatment recommendations for patients with Waldenström macroglobulinemia (WM) and related disorders: IWWM-7 consensus[J]. Blood.

[10] 陶 怡, 王 硕, 王 黎 (2021). 巨球蛋白血症患者临床特征和预后并与Pivotal研究比较[J]. 中华血液学杂志.

[11] Tam CS, Opat S, D'Sa S (2020). A randomized phase 3 trial of zanubrutinib vs ibrutinib in symptomatic Waldenström macroglobulinemia: the ASPEN study[J]. Blood.

[12] Dimopoulos M, Sanz RG, Lee HP (2020). Zanubrutinib for the treatment of MYD88 wild-type Waldenström macroglobulinemia: a substudy of the phase 3 ASPEN trial[J]. Blood Adv.

[13] Treon SP, Yang G, Hanzis C (2011). Attainment of complete/very good partial response following rituximab-based therapy is an important determinant to progression-free survival, and is impacted by polymorphisms in FCGR3A in Waldenstrom macroglobulinaemia[J]. Br J Haematol.

[14] An G, Zhou D, Cheng S (2021). A Phase II Trial of the Bruton Tyrosine-Kinase Inhibitor Zanubrutinib (BGB-3111) in Patients with Relapsed/Refractory Waldenström Macroglobulinemia[J]. Clin Cancer Res.

[15] 陶 怡, 潘 增凯, 王 硕 (2022). 不同方法和标本检测华氏巨球蛋白血症患者MYD88突变的初步探索[J]. 中华血液学杂志.

